# Tolerance, safety and accuracy of stress cardiovascular magnetic resonance in routine clinical practice

**DOI:** 10.1186/1532-429X-13-S1-P79

**Published:** 2011-02-02

**Authors:** Jeffrey Khoo, Benjamin J Grundy, Miroslav J Munclinger, Christopher D Steadman, Natalie Austin, Emer P Sonnex, Richard A Coulden, Gerald P McCann

**Affiliations:** 1 University Hospitals of Leicester NHS Trust, Leicester, UK

## Background/Aim

The use of stress cardiac magnetic resonance (CMR) as a clinical tool to evaluate myocardial ischaemia has increased significantly over recent years, but large-scale audit data is lacking. We therefore aimed to assess the tolerance, safety and accuracy of stress CMR in routine clinical practice.

## Methods

We retrospectively examined all stress CMR studies performed at our tertiary referral centre over a 20-month period, since the service was started in 2007. Patients were scanned in a 1.5T magnet (Avanto, Siemens), using a standardised protocol with routine imaging for late gadolinium enhancement (LGE). They were screened for contraindications to adenosine, and routine anti-anginal therapies, including beta-blockers, were not discontinued. Dobutamine stress was given in small number of patients in whom adenosine was contraindicated. Angiograms of patients who also had cardiac catheterization within 6 months of their CMR scan, were reassessed by an interventional cardiologist, blinded to the CMR data. For receiver-operator curve (ROC) analysis, CMR stress perfusion defects were graded into 5 categories (normal, probably normal, possibly abnormal, probably abnormal, abnormal).

## Results

A total of 654 patients were scanned. The mean age was 65 ± 29 years, and there were 63 inpatients (9.6%). The majority (639 patients; 97.7%) received intravenous adenosine (140mcg/kg/min for average of 3 minutes), 10 received intravenous dobutamine and 5 patients had both. Of the 15 patients who received dobutamine, 12 had no side effects/complications, 2 experienced nausea, and 1 chest tightness. Tolerance and safety data for all 644 patients who received adenosine are shown in Table 1.

**Table 1:** 

		No.	%
Minor symptoms (e.g. mild chest pain, breathlessness)		285	43
Number of patients where adenosine was discontinued prematurely		12	1.9
*Reasons:*	*Claustrophobia*	*4*	*0.6*
	*Significant hypotension*	*3*	*0.5*
	*Transient heart block*	*2*	*0.3*
	*Significant sinus bradycardia*	*1*	*0.2*
	*Bronchospasm*	*1*	*0.2*
	*Severe chest pain*	*1*	*0.2*
	*Scanner breakdown*	*1*	*0.2*
Transient Heart Block		5	0.8
Medical intervention (bronchodilators) needed		4	0.6
Hospitalisation		0	0
Myocardial Infarction or Death		0	0

**Figure 1:**
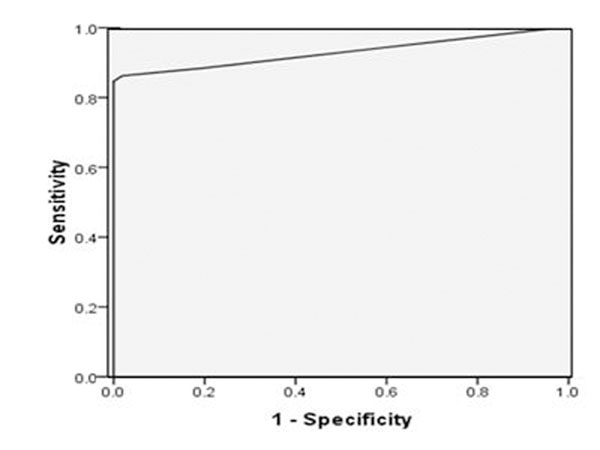
The area under curve (AUC) is 0.91± 0.02, with a prevalence of 71%. The overall sensitivity is 91%, specificity 86%, and accuracy 90%. These results compare very favourably with previous smaller research studies and meta-analyses.

241 patients also had coronary angiography. ROC analysis for detecting significant stenoses of >70% is shown in figure 1.

## Conclusion

We conclude that stress CMR, with adenosine as the main stress agent, is well-tolerated, safe and accurate in routine clinical practice.

